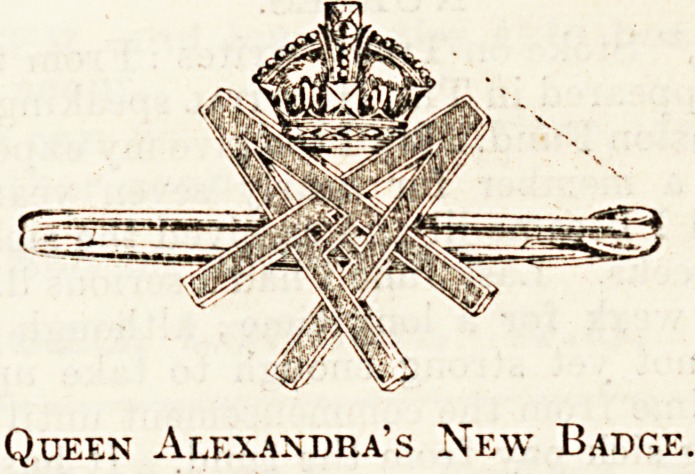# The Hospital. Nursing Section

**Published:** 1907-01-19

**Authors:** 


### The Hospital.

**Nursing Section.**

Contributions for "THE HOSPITAL," should be addressed to the EDITOR, "THE HOSPITAL" NURSING SECTION, 28 & 29 Southampton Street, Strand, London, W.C.

No. 1,062.—VOL. XLI. SATURDAY, JANUARY 19, 1907.

### Notes on News from the Nursing World.

### NURSES AND THE EARTHQUAKE IN JAMAICA.

THE announcement on Wednesday of the news that Kingston, the capital of Jamaica, had been visited by an earthquake on Monday afternoon was accompanied by an intimation that "the military hospital" had been burnt. But, as a matter of fact, there is no military hospital at Kingston, and neither members of Queen Alexandra's Imperial Military Nursing Service nor orderlies belonging to the Royal Army Medical Corps are serving in the ruined city. There is, however, a large general hospital, with a matron and a staff of about a score of nurses.

### THE MURDEROUS ATTACK ON A MEMBER OF THE NURSES' CO-OPERATION.

WE greatly regret the fact that Miss Fruzennah Low, an English nurse travelling abroad in pursuit of her duties, was murderously attacked on Saturday evening in the express from Turin to Paris, receiving such serious injuries that it is reported should she recover it will be three months before she can be moved. Miss Low was one of the most capable nurses attached to the Nurses' Co-operation in London. The assault, which was clearly perpetrated for the sake of plunder, was as dastardly as it was unexpected; and the sympathy of all nurses will be extended to the unfortunate victim of a miscreant's cupidity. Naturally enough the advocates of outdoor uniform urge that if she had been attired as a nurse it would probably not have been considered worth while to rob her. On the other hand, it may be said that it is quite unusual abroad for a nurse to wear uniform, and that if Miss Low had done so she would have attracted more attention than she desired.

### TEN PER CENT. OF NURSES OFF DUTY.

THE report presented to the Local Government Board by Mr. Jenner Fust and Dr. Fuller, who have been investigating the administration of the Salford Poor-law Hospital at Hope, proves the necessity for sweeping reforms. The inspectors found that the crib bedsteads in the male imbecile block are in a state of disrepair; that the male imbecile sick ward and padded rooms are not clean; that the cupboards are untidy; that the clothing and stores are kept anyhow and anywhere; that the laundry arrangements are inadequate; that the dietary requires revision; that the infirmary is materially understaffed; and, most serious of all, that the medical officer has rightly attributed much of the sickness among the nurses to the faulty condition of the drains. "In many cases," they add, "rat runs were observable, and rat holes were seen outside." As to the sickness of the nurses, they found that from October 1st, 1905, to October 1st, 1906, 24 nurses were off duty for 532 days, which gives on a total staff of 42 nurses a rate of 10 per cent. always off duty. When will the Local Government Board do its duty in the matter of this scandalous Poor-law Hospital, the condition of which is here shown to be a disgrace to Lancashire and, indeed, to the whole country.

### HER PATIENTS OR HER BOOKS?

No hospital matron ought to be placed in the predicament of having to choose whether she should neglect her patients or her books. This is, however, the position in which, according to the Chairman of the Board responsible for the government of the Guildford Isolation Hospital, the matron of that institution has lately been placed. It is satisfactory to learn that she did not neglect the patients. Of course, the books ought not to be neglected either, but though the matron admits that she agreed to keep them, she cannot be blamed because the number of patients in the Fever Hospital has for some time been so great as to require her undivided attention. The discussion at the last meeting of the Board reflects little credit on those who took part in it, and the same may be said of those who voted for a proposal, "that the matron be instructed to keep the books in future, and that if she finds it impossible to do so, she must pay out of her own pocket for them to be kept for her." The majority, however, objected to this ungenerous attitude, and adopted the report of the committee, who advised a small payment to a clerk who had posted the books up to date.

### CHANGES IN THE MILITARY NURSING SERVICE.

WE are officially informed that the following ladies have been appointed staff nurses in Queen Alexandra's Imperial Military Nursing Service: the Misses F. A. Loseby (Army Nursing Service Reserve), E. M. Lovell, C. Macrae, A. B. Nunn, A. Weir, H. M. Barnett, I. M. Johnston, and B. M. Nye. A number of changes have also been made in the service. Miss C. Hutton Potts, matron, has been transferred from the hospital at Middelburg, Transvaal, to the hospital at Standerton, South Africa. Sister S. Smyth has been transferred from Cambridge Hospital, Aldershot, to the Millbank Hospital; M. M. Bond to the Cambridge Hospital, Aldershot, from Netley; E. C. Cheetham from Curragh and K. A. Allsop from Devonport to the Herbert Hospital, Woolwich; A. Row to Devonport, from Portsmouth; A. Guthrie from Harrismith to Bloemfontein; R. Osborne from Bloemfontein, to Standerton; L. M. Lyall from Bloemfontein to Harrismith; A. L. Walker, from Aldershot to the ss. *Plassy* for Indian troopship service; M. Walker, from England, and F. M. MacGregor, from Middelburg, to Pretoria; and E. M. Denne from England to Bloemfontein. Staff Nurse G. S. Jacob has been transferred from the Connaught Hospital, Aldershot, to the Herbert Hospital, Woolwich; F. A. L. Smith, from York to Millbank; M. Barton, from Chatham to Devonport; E. K. Kaberry, from Millbank to Woolwich; A. S. Siddons, from Gibraltar to Netley; H. A. Hare, from Colchester to Devonport; M. Clements, from Colchester to Curragh; L. Belcher, from Woolwich, and S. N. Daly, from Hounslow, to Shorncliffe; M. German, from Gosport, A. A. Steer, from Millbank, and E. St. Quintin, from the Connaught Hospital, Aldershot, to Devonport. On appointment, Staff Nurses G. H. Sellar, B. M. Nye, and I. M. Johnston, have been sent to Netley; K. F. G. Skinner and A. B. Nunn to Aldershot; N. E. Smith, and E. H. Davies to York; C. C. M. Gibb to Portsmouth; C. H. MacCarthy and M. Ironside to Colchester; S. M. Wooler to Chatham; E. R. Collins to Hounslow; F. E. Morton and C. Macrae to Woolwich; K. F. Fawcett and L. A. Ephgrave to Shorncliffe; E. M. M. Malim to Gosport; A. Weir and N. Stewart to Devonport; H. M. Barnett and L. A. Burgess to Millbank; and F. A. Loseby (Army Nursing Service Reserve) to Bloemfontein. The promotions are: Staff Nurses K. A. Allsop, L. Belcher, C. T. Bilton, H. A. L. Jack, A. Rowe, D. J. Saunder, M. Clements, L. Cunningham, H. A. Hare, F. N. Roberts, F. A. L. Smith, and P. Steele, to be sisters; and Staff Nurses C. A. Coats, G. A. Aitchison, F. E. Manfield, A. M. Phillips, A. J. St. Clair, and D. M. Smith, have been confirmed in their appointments.

### ALLEGED DEARTH OF NURSES.

It is stated in one of the Manchester papers that there is "an extraordinary dearth of nurses in London." But the only evidence cited in support of the statement is that a particular institution advertised for a nurse and did not receive a single reply. As the name of the institution is not given, we are unable to estimate the value even of this proof. But two causes are suggested as the explanation of the alleged dearth. One is that the number of "medical institutions" increase more rapidly than the number of trained nurses—a hypothesis the accuracy of which we beg leave to doubt. Our contemporary goes on to say that much harm is done to the profession by such an incident as occurred the other day at West Ham Infirmary, where a nurse's eyesight was injured by the conduct of a typhoid fever patient." We have already expressed our regret at this incident, but it has no more to do with the point at issue than with the circulation of the paper in which it appeared. If there be any dearth of nurses, which we strongly doubt—to our knowledge an advertiser in our columns received 38 applications last week for a post offering no special inducements—it is not, we are convinced, because of an indisposition on any nurse's part to face the inevitable risks of a great calling.

### HACKNEY UNION INFIRMARY.

The Hackney Guardians have had under consideration a letter from the Hackney coroner forwarding the rider of the jury to their verdict, which we quoted a fortnight ago, but apparently it is not intended to make any addition to the nursing staff. The Chairman of the Infirmary Committee states that the Guardians have "adopted the system in general use in hospitals and infirmaries," and avers that "if they were to put two nurses for every one now on night duty they would increase their wages and bread and washing account by over £1,300 per annum." One suggestion was that in the unavoidable absence of the charge nurse from the ward a probationer should be available; and we are quite certain that in no well-regulated hospital or infirmary is a ward containing a number of sick persons left without a nurse during the night.

### THE STATE EXAMINATION IN NEW ZEALAND.

It appears from a report prepared by the Lady Superintendent of the Auckland District Hospital, relating to the State examination of nurses in New Zealand, that in December, 1905, 39 out of the 84 certificated nurses in that institution, or 46.4 per cent., were successful in passing this examination, as compared with a percentage of 37 successful candidates at the Wellington Hospital and a percentage of 3 at the Christchurch Hospital. It is not easy for English matrons to realise the difficulties under which the work of training nurses is carried on in New Zealand, or the beneficial effect exercised by the institution of an independent examination with a high standard. The percentage of successful candidates at the Auckland and Wellington Hospitals represents an amount of serious work on which the authorities are to be congratulated, and we have every expectation in the near future of seeing the figures considerably raised.

### COMPLAINTS AGAINST AN ISOLATION HOSPITAL MATRON.

The Winchcombe Rural District Council have had under consideration a series of complaints made by a former patient against the matron of the Isolation Hospital. Both the matron and the author of the complaints were present throughout the investigation, and it was wisely decided to admit the representatives of the Press. The most serious accusations were that the complainant's children were ill-treated by the matron when they were in the hospital; that while he himself was there she generally neglected her duties, and in various ways misbehaved herself. A practical answer to the first of these charges was that the complainant and his children are now in excellent health, which could scarcely have been the case if they had been badly treated at the hospital; and as to the others, they were denied *in toto*. The only witness called to support them was a servant at the institution, who had signed the papers written by the complainant. She confessed that she had never read them. On the other side, the solicitor for the matron, in his examination of the complainant, elicited replies of such a character that the Council, without deliberation, declared themselves satisfied that his charges were not proved, and ought not to have been made.

### INFIRMARY PATIENTS WITHOUT NURSES.

The King's Lynn Guardians have decided by a majority of 11 votes to 8 not to proceed with the appointment of an additional untrained nurse. This determination has been arrived at in face of the fact that, under existing conditions, the infirmary at Lynn is left every day for two hours and a half without any nurses. The Rev. A. H. Hayes, who, in quoting the fact, challenged contradiction and met with no response, said that it was impossible to avoid this with the present staff of nurses. But although he merely advocated the modest expedient of engaging an untrained nurse, he could not carry the majority with him. The majority appear to favour, as an alternative, that the night nurses should work 84 hours a week, and the day nurses for 76; and the probability of the serious consequences which may ensue owing to the absence of any nurse from the wards for two hours and a half does not seem to have impressed the Guardians. Yet King's Lynn is within a few miles of Sandringham!

### FEES FOR LECTURES.

The Carlisle Guardians, after considering the question a second time, have decided to allow the sum of fifteen guineas to be expended on lectures to the probationers at the Fusehill Workhouse. This is not an innovation, but there appears to have been doubts as to whether it was advisable to spend the amount again. The medical officer, however, explained that the work consisted not only in the preparation and delivery of the lectures, but in correcting and revising the probationers' notes, and, on the approach of the examinations, tendering further assistance. The course of training is for three years, and the lectures extend over six months in each year. At present there are but four probationers. The cost is well repaid by the higher class of women attracted to poor-law work in those Unions where, through the public spirit of the Guardians, adequate instruction is provided to probationers.

### THE NEW LOCAL GOVERNMENT BOARD ORDER.

We recently stated that an order had been issued by the Local Government Board with regard to the management of sick wards of Chelmsford Workhouse. As similar orders are in force at Farnham and Basingstoke Workhouses, and another is about to be issued to the Rotherham Guardians, the substance of the document may be interesting to some of our readers. There are two Articles, the first of which provides that it shall be the duty of the superintendent nurse to visit each of the sick and lying-in wards of the Workhouse daily, and to see that these wards have been duly cleansed and are properly warmed and ventilated. Further, that such arrangements are made as may be necessary for the proper care of and attendance upon the inmates both by day and during the night. According to Article II. the duty of making morning and nightly visits to the sick and lying-in wards of the workhouse is to cease to be part of the duties of the master or matron, as the case may be, though, with this exception, nothing in the Order is to affect the duties of the master or matron so far as these duties relate to the personal control of the workhouse.

### "MOTHER HILDA."

A few weeks ago we recorded the death, under the saddest possible circumstances, of a nurse who was refused admission to a religious order because it was not considered that she possessed the necessary qualities for the work. Mother Hilda was another devoted woman who in early life was occupied in nursing, but subsequently became the head of a religious organisation, a post which she filled to the satisfaction and benefit of all who were associated with her. This venerable lady, who was known as Mother Hilda Mary Stewart, of West Malling Abbey, recently passed away in the 78th year of her age. In her early days she was actively engaged in attending the sufferers from cholera during the epidemic, and continued nursing work as one of the sisters at St. Saviour's Hospital, Osnaburgh Street, London, until in 1868 the opportunity came for her to accept the Benedictine rule. She was appointed Superior of a religious community, first at Feltham, then at Twickenham, and finally in 1892 she went to West Malling Abbey.

### HOSPITAL AND HOME FOR INCURABLE CHILDREN.

On Tuesday, last week, the annual "At Home" of the Hospital and Home for Incurable Children was held at Northcourt, Hampstead, and was well attended. The large room was filled by three o'clock in the afternoon, when some of the children sang carols very prettily, Miss Duff, who has taken much trouble in training them, accompanying them on the harmonium. Considering all the disabilities from which the children suffer, the result did her great credit. After tea, a concert brought the proceedings to a satisfactory close. We hope that the strong support given to this year's "At Home" by local visitors presages additional contributions to the hospital, which is doing good work and is in need of further help.

### SHORT ITEMS.

The annual concerts for the nursing staff of St. George's Hospital were held in the Board-room last week, the concerts being personally arranged by Princess Victoria of Schleswig-Holstein.—Miss Rednall, who has been working on the staff of the Society of Chartered Nurses in London, has taken the Nursing Institution at Chelmsford. She was trained at the Royal Free Hospital, London.—The first Pound Day at the General Lying-in Hospital, York Road, London, was held on Tuesday last, when many friends and ex-nurses, either by post or personally, sent in gifts.

### The Nursing Outlook.

"From magnanimity, all fears above;
From nobler recompense, above applause,
Which owes to man's short outlook all its charm."

### THE HEALTH OF NURSES AND SICK PAY.

We have before us some interesting facts relating to the health of the working nurse. They include all classes of nurses, but largely those engaged in private work. The returns for 1,400 nurses show, over a period of years, that the average annual sickness is practically uniform in amount, and that about 18 per cent. of the nurses have been disabled each year by accident or illness. The percentage of severe cases is shown by the circumstance that 54 per cent. of the invalid nurses have suffered from illness disabling them for from five to 52 weeks in each year. Fourteen per cent. have suffered from more than one illness during the same year. More than 80 per cent. of the sick were attacked by acute specific disease; about 14 per cent. by diseases of the alimentary canal; some 13 per cent. by functional nervous disease; 13 per cent. were cases of accident and operation, and a similar number suffered from diseases of the respiratory organs, and from general disease and debility. It is not a little remarkable that the number of patients, year by year, in each class of disease, shows marked uniformity. We consider 18 per cent., in view of the character of a nurse's work, to be a surprisingly small percentage of illness amongst so large a body of nurses of different ages, discharging their duties in various branches of their profession, and so representing the average risk which every nurse has to incur. Still, when it is made clear, that one in five of the whole body of working nurses do in fact suffer, each year, from severe illness, which incapacitates them for work for several weeks, we hope no nurse of average intelligence will be likely to overlook the importance, in her own case, of underwriting this risk, by taking out a policy for sick pay in the Royal National Pension Fund for Nurses. It would be a useful and desirable plan, for the authorities of each training school, to keep a register of all cases of illness, which occur among the nurses each year, and to publish a few statistics embodying their experience in this matter.

No better service can be rendered to any nurse, by the authorities of her training school, than to impress upon her the importance of insuring against sickness, directly she receives her certificate of training. The earlier the nurse exhibits this measure of thrift, the greater will be her security, and the smaller will be the payment, which this necessary protection will cost her. There is a general idea amongst nurses, that, because they are young, they need have no care to provide against the day of sickness, because so many of them are robustly well. In practice, as the statistics and information show, sickness very frequently attacks the younger women, owing to the circumstance, that they have not become habituated to the atmosphere of sickness, and that they are apt to disregard certain precautions, when working on their own account, especially in regard to regular and continuous exercise, which renders them peculiarly liable to attack. In our view, the best of many good features of the Royal National Pension Fund for Nurses is, that, every nurse, who takes out a policy, if she enters for sick pay in the days when she is strong and well, can make provision for her whole life, so that her mind may be free from anxiety as to the future. So far as we have been able to ascertain, from some ten years' experience, one nurse out of every hundred, that is 1 per cent. of the whole body, become permanently incapacitated during the years of their working life. This fact, if it could be brought home to the mind of every nurse, would certainly make each worker alive to the importance of sick-pay provision. It becomes more and more necessary, to impress upon each nurse the importance of being insured against the day of sickness. If this could be brought clearly home, the number of those insured against sickness would increase rapidly each year.

We note that many nurses, who are working in institutions, feel that there is no need of sick pay provision in their case, because the authorities make arrangements for their treatment and care when ill. This is no doubt true, of the majority, at any rate, of the best administered institutions employing nurses, but when the period of convalescence arrives, those nurses who possess a sick pay policy are in a position to take an adequate holiday, in comfort, and to thereby, frequently, lessen the period of convalescence. It is essential that all working nurses, during their years of service, should, as far as possible, protect themselves against the loss and expense due to illness. This can readily be done by the exhibition of a minimum of thrift, providing a sick-pay policy is taken out by a nurse when she becomes certificated, because the younger the nurse the smaller is the premium which she will have to pay during each year of her working life. This fact, coupled with the point we have just made in regard to convalescence, should convince the educated nurse, at any rate, that the small sum she sets aside, each year, as a provision against illness, is the wisest expenditure she can make. The success of the Royal National Pension Fund has been remarkable and satisfactory. It has already brought great comfort and given great strength to working nurses as a body. Both these advantages would be greatly extended, if every working nurse had the wisdom to protect herself, by the possession of a sick-pay policy in the Pension Fund.

### The Care and Nursing of the Insane.

By Percy J. Baily, M.B., C.M.Edin., Medical Superintendent of Hanwell Asylum.

### II.—NURSING THE SICK.

(*Continued from page* 205.)

### 6. The Administration of Medicine.

The nurse must always bear in mind that all medicines are composed of more or less potent drugs which, when introduced into the blood, exert some sort of influence over the functions of the various organs of the body. Very many of them are powerful poisons, and of these naturally only small doses are ordered. Medicinal agents may be used to produce either a local effect upon the part to which they are applied or a general effect upon the whole system. In the latter case they must be introduced into the blood-stream.

The various channels by which drugs may be administered are: (1) The mouth; (2) the lungs; (3) the skin; (4) the bowel.

#### 1. *The Mouth.*

The vast majority of medicines are given by the mouth, are swallowed, and are absorbed into the blood from the stomach or upper part of the intestine. Either liquid or solid substances can be given in this way. The liquids are generally dispensed in the form of mixtures, but some strongly flavoured liquids, like turpentine or castor oil, may be administered in gelatine capsules. Oils like castor oil or cod-liver oil may be given as emulsions. Solid substances may be given by the mouth either in the form of pills, powders, or lozenges.

Mixtures are usually prescribed in bottles holding twelve or six ounces, and in both sizes there are as a rule twelve doses. Occasionally eight-ounce bottles are used containing either eight or sixteen doses. Before pouring the dose out the nurse should make a routine practice of carefully reading the directions on the bottle and the name of the patient. In this way only can mistakes which may lead to the most serious consequences be avoided. The instructions as to the time and method of administration must always be accurately carried out. The bottle should always be thoroughly shaken, even if this is not stated on the label. In pouring out the dose the bottle should be held with the label upwards, so that if a drop should cling to the lip of the bottle, it may afterwards run down the side without passing over and soiling the label. The dose must be carefully measured in a medicine measure, which should be held perfectly level. The quantity is usually expressed in spoonfuls, which have a certain definite meaning and may be written in symbols as follows:

Two tablespoonfuls equal one ounce (℥j.).
One teaspoonful equals half an ounce (℥ss.).
One teaspoonful equals one drachm (ʒj.).
One dessertspoonful equals two drachms (ʒij.).
A drop or minim is the 60th part of a drachm (♏j.).

It will thus be seen that one tablespoonful is four times as much as one teaspoonful, and that one ounce contains eight teaspoonfuls or drachms. When the dose is ordered twice daily it should be given at 10 A.M. and 5 P.M.; if three times daily at 10 A.M., 3 P.M., and 7 P.M.; if four times daily at 10 A.M., and 2, 6, and 10 P.M. Sometimes a dose is ordered to be taken three times daily before or after meals; in the former case it should be given about 15 or 20 minutes before food is taken, and in the latter case immediately after the meal is finished.

Certain forms of medicines are always given at particular times of the day. Sleeping draughts, for example, are given at bed-time or at the time that the patient is composed for the night and not likely to be again disturbed. Saline aperient draughts, seidlitz powders, and black draughts should always be given in the morning, at least an hour before the patient takes his morning meal. On the other hand, aperients which take some hours to act are usually administered at night, at or about bed-time. Drugs which are likely to upset the stomach and produce nausea are usually ordered after meals, as cod-liver oil or mixtures containing iron. Also all mixtures which contain arsenic are always given on a full stomach. Many more examples might be given, but enough has been said to show the importance of strictly carrying out the instructions given.

As soon as the dose has been administered the medicine measure should be carefully washed and the bottle well corked and locked away in a special cupboard. On no account should it be possible for the patient ever to reach the medicine. This is especially the case in dealing with lunatics who might thus find a ready means of attempting, perhaps successfully, to commit suicide.

Pills usually contain drugs which are either not readily soluble, and are therefore difficult to administer in the form of mixtures, or which have a very nauseous taste. Many patients have great difficulty in swallowing pills, this difficulty usually arising from nervousness. If the pill be placed on the tongue and a mouthful of water then taken and swallowed the pill will usually be swept down the throat, or it may be cut up into a coarse powder and mixed with a little jam, in which form it can usually be swallowed by the most fastidious person.

Powders may either be placed on the tongue and then washed down with a gulp of water, or they may be mixed with a little jam.

Lozenges frequently contain drugs which are intended to have some local effect upon the mucous membrane of the mouth or throat. They should be kept in the mouth until they are slowly dissolved by the action of the salivary juices. They may also, however, contain drugs which are intended to be absorbed into the blood, such as opium or ipecacuanha. Various local appliances may be made to the mucous membrane of the pharynx and mouth by means of sprays, syringes, and brushes.

#### *The Lungs.*

The only drugs which can easily be introduced into the blood through this channel must be in the form of gases or fluids which very rapidly evaporate, and which may therefore be considered as gases. These are used in order to produce anæsthesia (unconsciousness of pain) during various surgical operations. They include laughing gas, chloroform, ether, and some others.

Various volatile substances are, however, often breathed into the lungs in order to produce some local effect upon the mucous membrane of the throat and air passages. Such applications are called inhalations. Commonly the vehicle which is used is steam, the effect of the drug being frequently greatly enhanced by the heat and moisture which thus accompany it—or the drug may be inhaled as a very finely divided solution in the form of a spray; or, lastly, the inhalation may be in the form of fumes produced by ignition. There are many special forms of apparatus to be obtained for producing inhalations, but the simplest one is an ordinary jug, into which some boiling water is poured. If the steam is to be medicated a certain amount of the drug, according to instructions, is put into the water and the neck of the jug is surrounded with a folded towel. The patient then places his mouth and nose over the jug and inhales the rising vapour. The jug should be wrapped round with flannel in order that the contents may retain their heat as long as possible.

Briefly, the chief effects which are sought to be obtained by inhalations are to soothe irritable states of the mucous membrane in the early stages of inflammatory conditions, such as acute bronchitis, or to stimulate the bronchial secretion, and thus assist in the removal of expectoration in case of chronic bronchitis or to relieve spasm as in some forms of asthma.

### The Nurses' Clinic.

### THE DISTRICT NURSE AND EPILEPSY.

Except when epilepsy is complicated by some other complaint, the District Nurse is seldom called in for the express purpose of attending to an epileptic patient, but the disease is so sadly common among the poor that in every district of an average size she will certainly find in the houses she visits several persons suffering from it in its varying degrees. Epileptic seizures vary greatly in intensity, but still more in frequency. A man may be, strictly speaking, "subject to epilepsy," and yet the fits occur at such long intervals that they are unknown to his employers, and make no practical difference in the market value of his day's work; or the fits may be of sufficient frequency to confine him to certain classes of work, or it may be impossible for him to obtain any employment at all. In severe cases the fits may number from three to twenty or even more in the course of twenty-four hours. In some cases they occur regularly on certain days of the week, but as a rule there is great variability on this point.

The disease is said to attack females more frequently than males, but the district nurse's experience gives her exactly the opposite impression. The causes of epilepsy are not known, but the patient's history is usually one of delicate nervous organisation, overwork, harsh treatment, or severe mental shock. The symptoms are: ghastly pallor; a piercing shriek; the patient falls down as if shot; there is complete unconsciousness; the tongue is bitten; the limbs are violently jerked; in a few minutes the convulsions cease, the patient passes into a state resembling sleep, and when he rouses himself has no recollection of what has occurred. Patients generally feel a premonitory symptom known as an aura, usually described as being like a breath of cold air, or a "creepy" sensation accompanied by fear. Occasionally the aura is felt some hours before the attack, but often the patient has not time to lie down or to place himself out of danger. The average duration of a fit is two or three minutes, but they sometimes follow one another with no interval of consciousness, and this succession of fits is commonly described by the relatives as a single fit "lasting some hours."

Epilepsy is distinguished from hysteria by the single, blood-curdling shriek at the beginning, by the complete unconsciousness of the patient, and by his absolute recklessness as to all bodily injury, actual or possible. In feigned epilepsy the face is red instead of ghastly pale, the skin is hot, and the tongue is not bitten.

The treatment during a fit is to loosen the patient's clothing round the neck, lay him on the ground or on a large low couch, and place a cork or pad between his teeth to keep the tongue from injury. Attempts to control the convulsive movements must never be made, as if successful they might result in serious strain or lasting injury.

The general treatment required by epileptic patients is such as can rarely be obtained for them in the houses of the urban poor. They need kind, but firm management, and constant supervision; light, warm clothing, plain, nourishing food served with great regularity; separate beds, specially arranged and very little raised above the level of the floor; steady occupation; plenty of space, out-door exercise; fresh air night and day. How many of these conditions can be found in homes originally poor and still further impoverished by this heavy misfortune? One member of the household will over-indulge the patient, another be wantonly severe, while a third cannot be restrained from thwarting and teasing even at the imminent risk of bringing on a seizure. Training, food, clothes, bedding, all leave much to be desired. Suitable occupation is hard to provide, and amusement and change of scene become intensely difficult when school, Sunday school, church, chapel, gymnastics, the swimming bath, the Boys' Brigade, and "treats" of every description are inevitably cut off.

The nurse must do what she can to improve the general condition of the patients while they remain at home, but if practicable they should be removed to an epileptic hospital, or sent to live with relatives in the country where they can lead an outdoor life. It is not only on their own account that this complete change of environment is desirable. One of the saddest features of the disease is the mental and moral deterioration which invariably sets in when the fits are frequent and long continued. Every few months we are horror-struck by the report of some purposeless, inexplicable murder, generally of a young and helpless child, committed by a person well known to be an epileptic. Now this mania had not developed suddenly, but by degrees, plainly perceptible to a careful observer. I remember one good-looking, well-grown lad of fifteen, previously remarkable for what the poor call "tender-heartedness," who deliberately tried to stamp on the hands of his baby niece as she crawled about the floor. A few months later, although he belonged to a rigidly respectable family, he stole a pair of boots. The mother could not extort from him the name of the shop from which he had taken them, and surreptitiously dropped them in the street, overcome with shame and fear. Not long after he displayed tendencies which alarmed the

THE NURSES' CLINIC.—*Continued.*

parents still more, and they finally consented to send him to a Home where they paid 5s. a week towards his maintenance. They only fully realised the risk that they and their neighbours had been running when told that he was the worst and most hopeless case out of 400.

For the poor lad's personal benefit little or nothing could be done : he had been kept cooped up in a "block" for five years, scarcely ever going for a walk, and his general health was completely undermined. The nurse must do her best to persuade parents to part with the sufferer while the disease is still in a hopeful stage, pointing out to them that a sedentary life is deadly, and that the mental distress and humiliation that so much adds to the afflicted lad's sufferings will be greatly reduced when he finds that he is one of many, some better, some worse, and no longer a person to be stared at, or to feel that he is enduring exceptional hardships and restraint.

Cold and tepid baths may be of use, also stimulants in moderation. The teeth should be attended to, and—especially in young children—worms may be a source of irritation, or circumcision may be desirable.

The first attacks of epilepsy may be so slight as almost to escape notice : the sufferer looks very pale, suddenly leaves off whatever he is doing, but resumes his work or conversation a few seconds later only conscious of having experienced a slight feeling of giddiness.

In what is known as Jacksonian epilepsy the patient does not lose consciousness, and the convulsions are confined to a single part of the body. This form results from tumour, or injuries to the skull, and operation may result in complete recovery.

### Incidents in a Nurse's Life.

### A STRANGE STIPULATION.

I WAS attached to a private nursing institution in Scotland, situated in a sunny street in a busy town. The nurses who were in from cases could go out for a walk or do as they liked till sent for, as long as they remained in the house either during the forenoon or the afternoon.

"Nurse, you are wanted in the business-room," said a bright-looking nurse, putting her head in at the nurses' sitting-room door, about 3 P.M.

"Oh, dear!" I exclaimed, feeling the usual flutter at heart which a nurse always feels when she is to be sent she knows not where. "You're booked!" exclaimed the others, who were in the sitting-room busy with their work or books, knowing full well what the signal meant.

"Well, good-bye, girls, and good cases to you," I said, gathering up my work and running downstairs to matron's business-room.

Matron was to my mind an ideal woman, kind, gentle, and considerate, and taking a personal interest in each of her 150 nurses.

After I had knocked timidly at the door I found her in company with another lady, whom I recognised as a lady doctor. "This is Nurse M., Doctor," said matron in her gentle way, "and surely her eyes are blue enough!" and they both looked at me and laughed. "You are puzzled, I expect, as to what we are laughing at, nurse," said matron; "but Doctor requires a nurse with blue eyes, and as yours are very blue, in conjunction with your other qualifications, I thought you might suit. Whatever will people want next? I have been asked for old nurses, young nurses, fair nurses, and dark nurses, tall nurses, short nurses, etc., but this is the very first time I have been asked for a blue-eyed nurse. As far as eyes are concerned yours ought to do, so I will leave you to talk over the case with Doctor."

"Well, nurse," said the doctor, "there is not much real nursing at the case, and it is only for a few days I shall want you, to give an old lady a rest. She has tired herself out, attending to her husband who is blind, and they both have had bad colds, so I want her to stay in bed for a few days, and you can give a little attention to both of them. They are eccentric people, but very kind if you use a little tact with them. See that they take their meals, and you won't need to stay up all night. Go to your bed and get a good sleep if they keep you; for they were unwilling to have a nurse at all, but I insisted, and they can well afford it. I will call in the morning."

I got my box on to the top of a cab, and was off in a short time. We were often sent long journeys, but this was a town case, so the cab took me all the way. It was raining hard, and just as we were crossing a busy thoroughfare the horse slipped and fell right across the car lines. A crowd gathered in a minute, and I jumped out, one elderly lady remarking, "Dear me, nurse, how white you are." Indeed, I felt rather white, though I was vexed at showing my fears in my face, but I calmly rejoined, "Perhaps you would have been white, too, if you had been inside that cab." The horse by this time was helped up and once more into harness, so I got in again, and was soon landed at my patients' address. As the doctor had said, there was not much nursing to be done. Both husband and wife were queer and needed a lot of humouring and tact, but the old lady turned out kindness itself and was quite devoted to her blind old husband, who was also very good to me. I did what I could for them and for the household in general, and when I got to know them better I asked the reason of their wanting a blue-eyed nurse. I found that they were once very much deceived by a dark-eyed person and lost a large sum of money through him, and now not only trained nurses, but servants, clerks, and doctors—in fact, anyone who had any connection with them, was bound to have blue eyes, or they would not employ them. I returned to the home at the end of three very happy weeks, wearing a feather in my cap, for they had promised that if ever they were feeling ill they would have a trained nurse straight away—only, of course, she would need to have blue eyes.

### To Nurses.

**WE invite contributions from any of our readers, and shall be glad to pay for "Notes on News from the Nursing World," "Incidents in a Nurse's Life," or for articles describing nursing experiences at home or abroad dealing with any nursing question from an original point of view, according to length. The minimum payment is 5s. Contributions on topical subjects are specially welcome. Notices of appointments, letters, entertainments, presentations, and deaths are not paid for, but we are always glad to receive them. All rejected manuscripts are returned in due course, and all payments for manuscripts used are made as early as possible after the beginning of each quarter.**

### Illustrations of the Life of a Modern Nurse.

OFF DUTY AT GUY'S HOSPITAL.

IN ONE OF THE RECREATION ROOMS AT THE RAPHAEL NURSES' HOME, LONDON.

### A Severe Case of Rheumatic Fever.

### EXAMINATION QUESTIONS FOR NURSES.

The question was as follows: "What treatment should you apply (from a strictly nursing point of view only) to a severe case of rheumatic fever? N.B.—The answer must not touch upon drugs or anything to do with the medical man's province, but deal with the subjects of bed-making, washing, and dieting, if the latter is permitted by the doctor.

### First Prize.

The patient should wear flannel night attire, and must sleep between blankets. A twill pillow-case should be used in preference to a linen one; the same for the sheet, which may be placed over the blankets and turned in two or three inches at the top to prevent the rough surface coming in contact with patient's face.

Two or three blankets are generally sufficient, as many patients cannot bear the weight of much clothing. The bed should be carefully screened from draughts, and the temperature of the room kept at about 60° F.

The patient's joints should be swathed in cotton-wool and lightly bandaged, flannel bandages being used for preference.

The night-clothes should be changed at least once a day, as there is profuse sweating; have the clean one warmed and ready at hand, and change as quickly and gently as possible.

Do not expose the patient more than necessary when washing him. Each limb should be washed separately, and bandaged up again before the next is commenced. Have everything at hand, and plenty of warm towels, fresh wool, and bandages ready. It is advisable to keep two sets of bandages going, so as to avoid stopping to roll up those just removed for immediate use again. Be very careful in moving the patient, so as not to cause more pain than can be helped. ,, arm the bed-pan previous to giving it to the patient, or else cover round with flannel to prevent giving a sudden chill. Take patient's temperature every four hours, unless ordered otherwise. If the diet is left to the nurse's discretion, give fever diet—milk and light farinaceous food. Should all drinks be ordered warm, take the chill off the medicine by placing the glass in hot water; and then, after pouring in medicine, replace the glass in hot water for a minute.

Look out for, and report at once, any symptoms of heart complications, such as pericarditis, which may be suspected by vomiting, palpitations, and inability to lie down.

Do not allow the patient to make any sudden movement or exert himself in any way, and keep him very quiet. During convalescence continue to keep patient from over-exertion, and guard against draughts and sudden changes of temperature.

The patient must continue to wear woollen garments next the skin for a considerable time; in fact, he ought always to wear them.—Rosemary.

### Second Prize.

Put the patient between blankets in a well-ventilated room at an even temperature and free from draughts. Appetite may be enticed and strength maintained by giving small and frequent feeds of milk, milk puddings, thin mutton broth, etc. After the severity, foods rich in vegetable matter must be given, and no red meat without orders. Chicken and fish, perhaps, will be the first allowed, as they are less rich in animal matter. Barley-water flavoured with lemon, lemon juice, soda and milk, may be given to drink. A four-hourly chart must be kept. It is best to have four thin blankets for use, and a liberal supply of night attire. When one lot gets wet from the profuse sweating another lot must be ready dried and warmed to put on. Sponging the patient must be done very carefully under the blanket with warm water unless otherwise ordered; it must be done in sections, and any part that is too painful must be done as far as possible without moving, taking special care about the armpits, groins, and any parts where the skin touches. There is a very strong, sour smell from the sweating. A few drops of eau de Cologne added to the water will be refreshing and eliminate the smell. The pain in the joints is often very severe: they may be done up in cotton-wool and bandaged and rested (if ease can be obtained) on a pillow or a rubber hot-water bottle. A mackintosh must be placed under the bottom blanket. He must not be allowed to get out of bed, or even sit up, as the complications must be borne in mind. The throat may be gargled with a little weak solution of Condy's fluid, and the tongue and lips swabbed with a solution of boracic lotion. The bowels must be moved at least once a day. Epistaxis is often preent in rheumatic fever. Should it not stop with the usual methods of treatment, the doctor must be sent for, as it often requires plugging. The bed-clothing must be light and warm, and a cradle used to prevent the clothing touching the painful joints. Everything possible must be done to prevent chills and shock, as either of these may have a fatal ending.—Lanoitan.

### Good Answers This Month.

The papers sent in this time are much in advance of previous efforts, and, judging by the number sent in, the question must have been a popular one. "Rosemary" and "Lanoitan" are the successful candidates. Their papers are exceedingly good, but so are those of many other competitors; they both, however, notice small points which if attended to conduce to the sufferer's comfort. "Rosemary" speaks of placing a cotton sheet outside so that the rough blanket may not worry the patient's face, and mentions the common-sense fact that, having two sets of bandages, will obviate delay and consequent suffering. She also is practical about warming drinking vessels, which is sometimes ordered by doctors. "Lanoitan" has very good ideas about dieting, and he has a remedy for the distressing smell that comes from such patients, and draws attention to the care required in washing where two surfaces touch. Neither prize-winner speaks as they should do of the personal garment being slit up the back and the sleeves, the latter being fastened with tapes; nor do they emphasise the washing process sufficiently. It needs to be much more than sponging with "warm" water. What is needed is a thorough wash with plenty of soap and hot water. If the patient can bear it, this should be done twice a day; the soap, being alkaline, has a very beneficial effect on the sour smell.

### Honourable Mentions.

This is gained by "Vallis," "Miramie," "D. C. E.," "Shepperton," "Elsa," and "Ripona." "Vallis's" paper is very good, and her ideas on dieting are superior to those of all the other competitors.

### Question for January.

If ordered to strap a leg for the cure of an ulcer, how should you proceed? Describe your preparations, mode of procedure, and, finally, in what manner should you remove the strapping when necessary. This is a simple question; but beware! there are pitfalls for the unwary and the conceited! The Examiner.

### Rules.

The competition is open to all. Answers must not exceed 500 words, and must be written on one side of the paper only, without divisions, head lines, or marginal notes. The pseudonym, as well as the proper name and address, must be written on the *same* paper, and *not on a separate sheet.* Papers may be sent in for 15 days only from the day of the publication of the question. All illustrations strictly prohibited. Failure to comply with these rules will disqualify the candidate for competition. Prizes will be awarded for the best two answers. Papers to be sent to "The Editor," with "Examination" written on the left-hand corner of the envelope.

In addition to two prizes honourable mention cards will be awarded to those who have sent in exceptionally good papers.

N.B.—The decision of the Examiner is final, and no correspondence on the subject can be entertained.

Any competitor having gained three prizes within the current year shall be disqualified from taking another until 12 months shall have expired since the first prize was gained.

### The Babies of Wakefield.

A VERY interesting and important contribution to health literature is Miss Marguerite Boileau's report of her three years' work as health visitor under the auspices of the Wakefield and District Sanitary Aid Association. Miss Boileau seems to have been allowed very much of a free hand in the work to which she should devote herself, and, after having given six months to the completion of some statistics of working-class homes prepared by her predecessor, she came to the conclusion that the best thing to which she could devote her attention was the welfare of infants. Like other observers, she had noted that the great decrease in the national death-rate during the last forty years did not seem to have affected that of infants under one year of age. Indeed, the highest proportion of infant deaths recorded in the statistics of the last century was in 1899, when it reached the alarming figure of 163 deaths for every 1,000 births. Some people are inclined to view a high infantile death-rate with indifference, if not with complacency, arguing that the children who die are weaklings, and that it is to the advantage of the race as a whole that these sickly ones should not cumber the ground. But Miss Boileau has two arguments against this convenient theory. "Medical opinion," she says, "gives 8 to 9 per cent. as the average proportion of babies born weakly; an infant death-rate of 80 or 90 is inevitable, but anything over 100 is preventable, and ought to be prevented. The surplus is entirely due to faults in tending and surroundings." As the rate of infantile mortality in Wakefield during the years 1893-1902 averaged 173 per 1,000, this estimate—which is not a fancy one, but founded on reliable facts and statistics—forms in itself a serious indictment of Wakefield methods and Wakefield mothers.

But even more important from the national point of view is the case of those who survive. To quote Miss Boileau again: "The same conditions that weed out the weakly babies ruin the physique of the strong." If, for example, a mother is obliged to keep the baby in a damp cellar-kitchen while she does her daily work, bronchitis sets in, then pneumonia; if delicate, the baby dies—is "weeded out"; if strong it survives, but it will be with an enfeebled breathing apparatus, which in later years will convert an attack of whooping-cough, measles, or influenza into a dangerous illness, and make ready another victim for consumption. Therefore, the most important part of a health-visitor's work will not show in immediate statistics. However satisfactory in itself a lowered rate of infantile mortality may be, one should regard it chiefly as indicating that, while the weakly are preserved in life, the strong are preserved in health.

Miss Boileau does not agree with all the authorities regarding the causes of infant mortality. Two of the causes most commonly mentioned are the employment of married women in the factories and the decline of breast-feeding. As has been seen, Wakefield has a high infant death-rate. Yet Miss Boileau says that in Wakefield mothers of young babies do not go to work in the mills. "I have only had fifteen cases in over 1,000 in which the mother has worked in a mill, and only one of these babies was left while under three months old." Again: "To give the actual figures, I have found 6 per cent. of the mothers at work; over a quarter of these were unmarried women; in the remaining cases the reason was that the husband was receiving a very low wage, out of work, delicate, or a drunkard." Moreover, the work which these women do is not mill work, but washing or charing—that is to say, it is irregular, and does not involve such long hours away from home as factory labour, and Miss Boileau does not think that it is a factor in the infant mortality in Wakefield.

With regard to breast-feeding, Miss Boileau found that 56 per cent. of the infants that came under her notice had the breast only, while 12 per cent. had both breast and bottle, and 7 per cent. were weaned before the ninth month. As a matter of fact women of the working class consider breast-feeding much less troublesome than hand-rearing, and as they have no one to whom to hand over the charge of the child, the latter course has no advantages for them. Only 14 per cent. of the mothers were totally unable to suckle their children, and Miss Boileau thinks that a considerable proportion even of these would have been able to rear their children at the breast if they themselves had had sufficient nourishment and proper rest after the birth of the child. Miss Boileau gives full credit to the work done by crèches and milk depôts, but she points out that hitherto attention has been given exclusively to agencies for the betterment of the bottle-fed baby, and adds that there is as much to do for the breast-fed infant. "I have more often," she says, "to exert all my powers of persuasion to induce a mother with ample breast-milk to cease ruining her child's digestion by cramming it with bread-sops than I have to get a tubeless bottle into use in place of the murderous long tube."

The inference is that the cause of excessive infant mortality, at least in Wakefield—and who shall say that that town is exceptional?—is the ignorance of mothers of the commonest laws of health. Miss Boileau found that indeed there were worse people than the mothers in this respect—namely, the grandmothers, who were equally ignorant and far more obstinate. Improper feeding is a common cause of sickness and death. Miss Boileau mentions tea, gruel, bread, soup, arrowroot biscuits, rhubarb pie and pancakes, as among the things given to young children within her knowledge. In one case an eighteen-year-old mother had been advised "to give the child a taste of everything she took, and then her milk would never disagree with the baby." Obeying this dictum, she gave a four-days-old baby some tomato! Even when the child gets nothing but breast-milk, it does not follow that it is being wisely fed. It is a common practice of mothers to feed the child whenever it cries. Its cries may be the result of over-feeding, or of discomfort in clothing, or cold, or any other cause, but the remedy is always the same—another meal. This is the fault of quite well-meaning mothers. One woman told Miss Boileau that she meant to begin to give her baby solid food, because he was so cross all morning, that she thought that the breast-milk did not satisfy him. This was a model dame, so "house-proud" that she cleaned up her house before she attended to the baby, who was sometimes not washed until after dinner. When the child cried it never struck the mother that he wanted clean clothes and a bath—her only idea was to stodge him up with starchy food. In such instances the kindly interest and advice of a tactful lady health visitor is invaluable. Miss Boileau found that the regular weighing of the baby acted as an admirable object-lesson not only to the mother, but to the father, and she found this "the best peg on which to hang instruction as to the mother's need for food, rest and care when suckling her baby."

On this point of the mother's needs Miss Boileau has wise words to utter. Very seldom have any savings been put by for the inevitable expenses that the baby brings with it, and only after it is born does the mother begin to economise—generally beginning with her own food, says Miss Boileau, who adds:—"Privation and overwork at this time are the commonest causes of the loss of the breast milk." To meet these cases a provident scheme is being tried, which has already had good results. This is a kind of charity of which we have too little in England, but it may be hoped that the attention which is at present being given to the question of the birth-rate may arouse people to the fact that a healthy and well-nourished mother is a necessary factor if we are to have healthy and well-nourished children.

### Nursing at an Indian Hill School.

Here on the Himalayas, with exquisite mountain scenery and the eternal snows ever in sight, there is a school for English boys of the better class, with a Cambridge man who has taken Holy Orders as its Rector, and an English university staff, also graduates of either Oxford or Cambridge. The building is a very imposing one, though internally lacking in much that Europeans are accustomed to. We have also a church, a hospital with isolation ward attached, a gymnasium, a large library, and even a tuck-shop, in the compound.

We are two miles away from the station, and, standing on a great height, are supposed to escape many of the epidemics and minor illnesses to which the station inhabitants are subject.

Recently there has been a tremendous smallpox scare, several Europeans having died. Of course, it originated in the native quarter, and two early fatal cases of Europeans were those of missionaries who lived in or near that locality. Natives were vaccinated by the thousand, but the lymph first used was bad—*i.e.* ineffectual. For some time before the end of term our pupils were forbidden to go beyond the school boundaries.

The school being principally for the benefit of Indian-born children whose homes are in the torrid plains, there is naturally a good deal of sickness to combat; indeed, many of them are sent here more for health than for anything else. They vary in numbers from 100 to 120. We have a complete term of nine months, commencing in March and finishing late in November, the winter months being considered too cold for these children. The school is then closed, and we all welcome the vacation, as during the term it is one long grind for the staff. I have charge of the hospital attached to the school. These country-born boys are a wearying, wearing crew, not having the stamina of those born in Europe, always accustomed to being waited on, and noisy and mischievous beyond words.

To start with, when being brought up in the train under the charge of the masters, one boy very nearly lost an eye owing to being struck by a stone thrown by some Babus—natives of Bengal—in return for a similar attention on the boy's part. I opened my new packets of dressings and lotions for him on the very day of our arrival. All the boys were subsequently examined by the civil surgeon for any latent disease.

Many coming from warmer places immediately developed coughs and colds, which the surgeon always treated with gargles and throat-painting—troublesome, but effectual. Then one was always on the watch for malaria, which many of the youngsters had in their systems. I several times had a persistent temperature of 104 degrees to cope with. I used to give stock fever mixture of salicylate with saline purge, or, what was usually a better febrifuge, phenacetin with brandy—of course by doctor's orders. Then when fever was normal, or nearly so, quinine was given for a week, followed by a tonic of citrate of quinine and iron.

We were not troubled much with what is known as hill diarrhœa, but had a few cases, which promptly yielded to castor-oil emulsion.

One dear little lad developed acute appendicitis, supposed to be due to eating a potato-puff made with pastry. But there were others who said that, in fighting with another boy, he received a kick in the abdomen. He was removed to the Sanatorium and operated on by our very clever civil surgeon, but succumbed in twenty-four hours.

Later, a boy swallowed a key of no inconsiderable dimensions, being an ordinary-sized cupboard key. In spite of prompt treatment it was never again heard of.

Another, quite a small child barely nine years old, sampled an eight-anna piece (the size of an English shilling). This was subsequently presented to him, and he was delighted at regaining his week's pocket-money.

Another pupil put a piece of lead pencil into his eye, and yet another, one of the older boys, nearly lost the sight of one eye while fencing in the gymnasium owing to the slipping of a foil.

Accidents during hockey and cricket were frequent, as the school often played teams of Tommies, who were very rough for such young lads. In all these accidents there was much more tendency to fainting and collapse than among adult patients. Neither was there any doctor to be easily got at, as he only paid a short visit once a week.

Then there was a case of measles, quickly isolated and attended to by me. We lived in constant dread of a repetition for a little time, but no one else fell a victim. I had also one case of whooping-cough with bronchitis. We did not like to send the boy home, as his people were poor, and there were several brothers and sisters at home. We had also acute rheumatism more than once—these and fever occurring mostly during the rainy season. We have the heaviest rainfall of all India, commencing in early June and continuing till late September. We were considered to have got off comparatively lightly this year; but to a newcomer like myself the continual rain seemed dreadful.

Then, when we thought we were getting nicely over all our troubles, we had an outbreak of dhobies (Washerman's) itch, only known to happy India. There was a good deal of it in the station at the time. At the school it started with ringworm, said to be one and the same thing. Not the usual home kind, but on the body. They were isolated and well in a week with chrysophanic acid. Then there appeared cases with sores; we had them in hospital, and treated them with sulphur ointment one in four (1 in 4), and got them well. But as fast as you could fumigate and get rid of one batch in would come another, till at last we had the native doctor up to examine all. The native servants, with their wives and families, and the civil surgeon overhauled all the boys. There were twenty-seven cases in all, some with ever such slight spots being looked upon as suspects. They were all put into one dormitory, and only allowed to go to school wearing gloves and long washing-sleeves up their arms, and were smeared twice daily with sulphur ointment.

The gloves were also used for playing hockey and cricket, so naturally they were quickly filthy and in holes, also often lost and had to be replaced. There were two accidents and two ringworm cases admitted into hospital the last week of term. At last, however, they all finally departed in great glee under the escort of two of the masters, many with from three to five days' railway journey before them. The distances are so great in India, and some of the boys have to be shipped off to the Straits or Burmah. On the eve of departure they smashed thirty odd windows before leaving.

I do my own dispensing, which is considerable, as the local chemist is too dear, and one has to provide for the native servants and employés, as well as the household. So with no assistant it will be seen that my post is no sinecure. I have also been busy lately learning Hindustani under a native tutor. It is a disagreeable necessity and an expensive one; but I am glad I undertook the language now, as it saves me a lot of irritation and bad temper, English-speaking servants being rare in India.

Just now the mountains are at their loveliest. At sunset rosy lights are thrown over the distant snows. During the daytime it is fairly warm in the sun, but in the mornings there is a slight frost and it is cold enough for fires in the evening.

### An English Nurse Assaulted on a Foreign Railway.

On Saturday evening an English lady travelling in the express from Turin to Paris was murderously attacked and robbed. On the arrival of the train at the last Italian station before the frontier she was found in a first-class compartment half insensible, and was afterwards conveyed to Chambery Hospital. Her condition being very serious, trepanning was immediately performed, and when the operation was over she was able to make a statement through an interpreter. She is reported to have said that she was returning from Genoa on her way to Paris, where Madame Lechet expected her. At Turin a young man, fair-haired, with a moustache, and very stylishly dressed, entered her compartment. He settled down comfortably, and seemed to go to sleep. The nurse did the same, and she was in a deep sleep, when she received a violent blow on the head. She opened her eyes and saw the young man before her with a hammer in his hand. Before she had time to utter a cry she received a second blow on the head, and fainted. When she recovered she found that she had been robbed, her jewels and her pocket-book containing money having disappeared.

Subsequently it was ascertained that the unfortunate lady was Miss Fruzennah Low, a trained English nurse, residing in London, and that the Madame Lechet mentioned was a personal friend, who had made her acquaintance two years ago, when Miss Low was on the staff at a military hospital in England. Further inquiries have elicited the fact that Miss Low is a member of the Nurses' Co-operation, and, as Mrs. Lucas, the matron of that institution, informed our representative, was at St. Thomas's Hospital some time ago, working in the same ward as herself. She was trained at Charing Cross Hospital. It was a little more than ten years ago when Miss Low first joined the Nurses' Co-operation, and going to South Africa when the war broke out, remained there for two years, her services being much appreciated by the soldiers. She then returned to London and resumed work as a private nurse. In the middle of December last she left New Cavendish Street to take a lady patient to Bordighera, and on January 7 this year she wrote to Mrs. Lucas that she was returning, but asked for a holiday. The request was complied with, and she proposed to spend it with Madame Lechet in Paris. Mrs. Lucas thinks that the circumstance that she was travelling first-class may have been due to her patient, as the Co-operation only allow their nurses second-class. As soon as her identity was published in the newspapers on Tuesday telegrams kept coming in from former patients and friends inquiring about her, and the nurses of the Co-operation are exceedingly distressed. Mrs. Lucas, who states that she was one of the most capable nurses belonging to the Co-operation, despatched a nurse—one who had worked with Miss Low in South Africa—late on Monday evening to Chambery to take care of her and do all that is possible for her. The nurse telegraphs that on Tuesday Miss Low passed a good night.

### Queen Victoria's Jubilee Institute for Nurses.

Miss Janet E. Mundy has been appointed assistant superintendent, Gloucestershire County Nursing Association. Miss Agnes C. Angus has been transferred to Frizington from Sunderland, Miss C. M. N. Bell to Hertford from Woolton, Miss Edith M. Buller to Oxford from Rottingdean, Miss Mary T. Cunningham to Esclusham from Frizington, Miss Ellen L. Wells to Penzance from Gosport, and Miss Jane McEwen Hutchinson to Willington from Pateley Bridge. Miss E. Inston has been appointed to Crook, Miss Sarah E. Lebart to Woolton, and Miss Alice M. Goodman to Gosport.

### The Royal National Pension Fund.

THE NEW BADGE FOR THE QUEEN'S OWN NURSES.

The following illustration will enable our readers to realise how effective and really attractive the new badge is which her Majesty Queen Alexandra, the President of the Fund, has approved.

Queen Alexandra's New Badge.

The price of each brooch as shown in the illustration is eleven shillings and sevenpence in gold and red enamel, not one shilling and sevenpence as erroneously stated last week, and one shilling and one penny in gilt and red enamel, postage included. The new brooches may be obtained from the secretary at the office, 28 Finsbury Pavement, E.C., envelopes being marked "Brooch." We may add that the brooch is to be worn habitually by policy-holders of the Pension Fund, who are to reserve the armlet for use upon ceremonial and public occasions only.

### Appointments.

Borough of Burslem Infectious Diseases Hospital.—Miss B. S. Ashman has been appointed charge nurse. She was trained at Crumpsall Infirmary, and has since been assistant nurse at the City Hospital, Grafton Street, Liverpool, and charge nurse at the Isolation Hospital, Menston-in-Wharfedale.

British Hospital, Port Said.—Miss J. Davidson has been appointed charge nurse. She was trained at Crumpsall Infirmary, and has since been district nurse at Lumley, near Chester-le-Street. She holds the certificate of the Central Midwives Board.

Chippenham Union Infirmary.—Miss M. C. Munro has been appointed superintendent nurse. She was trained at St. Olave's Infirmary and the East End Mothers' Home, Commercial Road, London. She has since been maternity nurse at St. Mary's (Islington) Workhouse. She has also done private nursing.

Fletcher Convalescent Home, Cromer.—Miss Edith Adeline Noar has been appointed matron. She was trained at St. Bartholomew's Hospital, London, where she has since been night superintendent. She has also been assistant matron of St. Bartholomew's Convalescent Home at Swanley, Kent.

Liverpool Royal Infirmary.—Miss Isabel Callaghan has been appointed assistant lady superintendent. She was trained at St. Bartholomew's Hospital, London, and has since been sister at the Royal Hospital for Sick Children, Edinburgh, and night superintendent at the Royal Hospital for Diseases of the Chest, City Road, London.

Redhill Cottage Hospital.—Miss Ada Stuart Daniels has been appointed staff nurse. She was trained at the Royal Free Hospital, London, where she has since been theatre nurse. She has also been charge nurse at the South-Western Fever Hospital, London, and has done private nursing for the Mildmay Institute.

Salford Union Infirmary Hospital, near Eccles.—Miss Mary Pickup has been appointed charge sister. She was trained at Prescot Union Infirmary.

### Everybody's Opinion.

[Correspondence on all subjects is invited, but we cannot in any way be responsible for the opinions expressed by our correspondents. No communication can be entertained if the name and address of the correspondent are not given as a guarantee of good faith, but not necessarily for publication. All correspondents should write on one side of the paper only.]

### THE ROYAL NATIONAL PENSION FUND FOR NURSES.

"E. M. G.," Stoke-on-Trent, writes : From time to time letters have appeared in The Hospital speaking of the benefits of the Pension Fund. May I too give my experience of it? I have been a member for nearly seven years. Shortly after I joined I became ill and received the sickness allowance for six weeks. Last June I had a serious illness, which left me very weak for a long time; although I am much better I am not yet strong enough to take up my work. During this time from the commencement until now I have been receiving sick pay from the fund. It has come regularly, and has been a great boon. Does not this speak for itself? Will you, through The Hospital, allow me to express my grateful appreciation to the committee and the secretary, Mr. Dick, for their most kind consideration? I look upon the Pension Fund as the greatest boon to a nurse, for not only is it a splendid investment for old age, but it means a great deal to be sure of receiving an allowance when one is incapacitated. I hope this letter may influence some nurses who think of joining, yet hesitate. I can only say it was the best thing I ever did when I joined the Pension Fund.

### COTTAGE HOSPITALS AND STAFF NURSES.

"E. L." writes : Having had some experience as a staff nurse in cottage hospitals, I should like to say a few words in favour of these posts. Doubtless every nurse begins by entertaining and cherishing a desire to become a matron. This is necessarily the summit of her ambition, and perhaps it is just as well to aim high. Still, there are a few, more or less, humble souls amongst us, and by and by we may acknowledge, however reluctantly, that perhaps after all we are not equipped by Nature with, nor do we seem altogether able to acquire, the qualities which go to the making of a successful administrator of a large and important institution. When we have reached this stage our thoughts turn to fever, special and cottage hospitals, and to convalescent homes. Although at the present time the recognised method of attaining to one of these smaller prizes of the profession seems to be by the route of "sister," still, the post of staff nurse supplies valuable experience and preparation for these nurse-matronships, and I think that every nurse must know of instances where staff nurses have been successful in obtaining these appointments, probably in many cases because of their knowledge of cottage hospital work. Indeed, there can be no better training for the matron of a small institution than that obtainable by the senior staff nurse in a cottage hospital. A three years' certificate is a *sine qua non*, and the work though limited in quantity is good in quality, surgical cases preponderating; and it is by no means uncommon for critical operations of a rare nature to be undertaken in quite small hospitals. As there is usually no house surgeon the matron reigns supreme, and all responsibility devolves on her, but if the nurse proves herself capable and trustworthy and is a cheerful and willing worker she will find that the matron will confer with her on many points and will give her opinions due consideration, treating her in all respects as a valued colleague. She will, in fact, be the matron's understudy, taking her place during her absence and probably doing matron's duty in case of sickness or holiday. In this way she will gain confidence in herself and experience in housekeeping and in household management, which experience will stand her in good stead when she in her turn has charge of a hospital, and it will also count in her favour when applying for a post. The most attractive feature of cottage hospital work, however, lies in the more homelike character of the conditions of life. The rules so necessary to the effective working of a large institution are out of place here, or are so greatly modified as to be unrecognisable. After the rush of a large hospital a nurse once more finds herself, and is encouraged to be herself. In place of being merely cases the patients are individuals, with idiosyncrasies and individual hopes and fears, and the nurse, if she is a true woman, enters into their interests and sympathises with them in their troubles and joys. Among the minor attractions may be placed the fact that the small hospitals are very often beautifully situated either in or near to lovely country; and although it may be a weakness still there are persons who require in their life and work the help which Nature gives to those who love her. Just as some can scarcely exist away from the noise of the Strand, so others are drawn to a country life. Some of the best women and best nurses I have known who, if compelled to live and work in a large town, would break down altogether, are doing good and valuable work in cottage hospitals.

### AN INCIDENT IN A POOR-LAW INFIRMARY.

"Responsible" writes : After reading "an incident in a poor-law infirmary" in your issue of January 5, I should like to give you a slight idea of the way in which the night duty is arranged in a poor-law infirmary in Warwickshire. The building consists of two floors; there are two wards containing fourteen beds in each, with a small ward opening out of the large one on the ground floor. The top floor is on a similar plan. There are at the present time sixty-two patients; of these seven on the female side are mental cases. There is one nurse on duty from 8 p.m. till 8 a.m. She is held responsible for all patients in the building, and it is only in the event of a patient being in a dying condition that any extra help is given; then it is only an inmate from the workhouse who is sent over to sit with the patient, who, of course, being quite ignorant of sick nursing, is of not much help to the nurse. A few nights ago a female lunatic got out of the ward, and I was only just in time to prevent her from entering the male ward; she became very violent on being stopped, and, though ninety-one years of age, I had great difficulty in getting her even as far as the duty room, which is half way between the two wards on the top floor, and then was obliged to call up a patient and send her for the superintendent nurse. The next night when I came on duty I asked for a woman to sit with the patient and was refused by the superintendent nurse. I then appealed to the Master, who refused to have anything to do with the matter, saying that the superintendent nurse was head of the infirmary and responsible for all that happened in it. The following night whilst I was at the other end of the building downstairs the patient got out of bed and made up the ward fire and poked out a large piece of live coal, and was looking round the ward for a piece of paper to put it on with again. Fortunately, before she could find any, one of the other patients awoke and got her back into bed, and thus averted what might have been a very serious catastrophe. I may add that there are two assistant nurses here, and they take night duty every alternate month. I brought this case before the Board of Guardians at their usual meeting, with other matters of equal importance relating to the nursing here, but no notice whatever was taken of my complaint. I have therefore sent in my resignation after being here only six months.

### Death in our Ranks.

We regret to hear of the death on Friday last, after a short illness, of Miss May M'Kenna, staff nurse in Queen Alexandra's Imperial Military Nursing Service. The interment took place on Monday at Kensal Green Cemetery.

### Wants and Workers.

Miss Alice Maud, of Hollow Dene, Wellwood, Torquay, writes : I have a lot of nursing and medical books to dispose of, and a new cloak, a wallet, and hypodermic syringe. Do you know of any poor nurse who would like to have them? There are about a dozen books—Taylor's "Medicine," Walsham's "Surgery," Huxley's "Physiology," one or two on midwifery, etc., and I do not need them any longer, so should be glad to give them away to some poor nurse. I cannot offer to pay carriage.

### Notes and Queries.

**REGULATIONS.**

**The Editor is always willing to answer in this column, without any fee, all reasonable questions, as soon as possible.**

**But the following rules must be carefully observed.**

**1. Every communication must be accompanied by the name and address of the writer.**

**2. The question must always bear upon nursing, directly or indirectly.**

**If an answer is required by letter a fee of half-a-crown must be enclosed with the note containing the inquiry.**

*Dispensary.*

(173) I have the Apothecaries' Hall certificate for dispensing, and I desire to secure a temporary or permanent post. I have advertised in the medical papers, but they are so very expensive. Would it be any use advertising in THE HOSPITAL? I have held several temporary posts.—*Salisbury.*

As you have had several posts, could not your employers assist you? There is not a very large demand for dispensers. THE HOSPITAL is regarded as a first-class medium for such vacancies as yours, but we cannot advise you on this point.

*Qualifications of a Training School.*

(174) Can you give me information with regard to the qualifications of a certain hospital as a training school for nurses?—*Argon.*

In seeking a good training school for nurses it is of the highest importance to ascertain whether the nurses receive regular instruction, and ultimately a three-years' certificate. This information can be obtained by writing to the matron of any hospital selected. The number of beds should not be less than 100. The hospital you mention contains only 65.

*Workhouse Nursing.*

(175) I have had three months' training at a nurses' home and have worked for 10 years for a Cottage Nursing Association. Would this be sufficient experience for a workhouse nurse?—*A Constant Reader.*

Unless you became a probationer and started afresh, you would not be received at the larger workhouse infirmaries; but at some of the smaller ones you might possibly find work at a small salary.

*Home of Rest for Nurses.*

(176) I have stayed at the house of a nurse in Hastings, and I have been so comfortable I should like to recommend this nurses' establishment to others. She would be glad of a blind patient.—*Private Nurse.*

We regret we cannot recommend private establishments, but why does not the nurse advertise? Many nurses might be glad to hear of a comfortable home by the sea.

*Lending Library.*

(177) Can you give me the address of a free lending library in Somerset?—*Taunton.*

There is a lending library especially for nurses in Somersetshire. Do you refer to this? If so, write to Miss F. Joseph, Woodlands, Holford Bridgwater.

*District Nursing.*

(178) Can you tell me if I can obtain a post as district nurse, not holding the Central Midwives Board certificate? I have just finished my training.—*Leamington.*

As you write from a general hospital, are we to understand that you have just completed your training as a nurse, and not as a district nurse? If so, you will still have to train for district work. It would be best for you to write to the General Superintendent, Queen Victoria Jubilee Institute, 120 Victoria Street, S.W. If you have trained, you must advertise, or you might apply to some of the district nurses' associations, of which there is a list in "How to Become a Nurse" to be had, price 2s. 4d. post free, from The Scientific Press, 28 and 29 Southampton Street, Strand, London, W.C.

**Handbooks for Nurses.**

| | Post Free. |
|---|---|
| "How to Become a Nurse: How and Where to Train." | 2s. 4d. |
| "Nursing: its Theory and Practice." (Lewis.) ... | 3s. 6d. |
| "Nurses' Pronouncing Dictionary of Medical Terms." | 2s. 6d. |
| "Complete Handbook of Midwifery." (Watson.) ... | 6s. 4d. |
| "Preparation for Operation in Private Houses." ... | 0s. 6d. |

Of all booksellers or of The Scientific Press, Limited, 28 & 29 Southampton Street, Strand, London, W.C.

### For Reading to the Sick.

"THY WILL BE DONE."

WE see not, know not; all our way
Is night—with Thee alone is day:
From out the torrent's troubled drift,
Above the storm our prayers we lift,
Thy will be done!

We take with solemn thankfulness
Our burden up, nor ask it less,
And count it joy that even we
May suffer, serve, or wait for Thee,
Whose will be done!

*J. G. Whittier.*

Oh, I would speak this day to any who are in sorrow, or trouble, or affliction, and say, If your Master "learned obedience," and was "made perfect through suffering," will you refuse to share His burden, if you may afterwards win His crown? Will you put away from you by murmurings and discontent the rich blessing your suffering came to offer you? How well does our Church speak to all her suffering members in the office of the Visitation of the Sick, saying, "There should be no greater comfort to Christian men than to be made like unto Christ by suffering patiently adversities, troubles, and sicknesses. For He Himself went not up to joy, but first He suffered pain; He entered not into His glory before He was crucified. So truly our way to eternal joy is to suffer here with Christ, that we may rise again from death, and dwell with Him in everlasting life." . . .—*Bishop Walsham How.*

He Who suffered in our nature knows what our suffering is. Although He may seem to leave us for awhile in difficulty, nevertheless He takes no delight in such distress. Quickly does He welcome the gift of love which the heart in its anguish offers to Him. If we fail to love Him, and doubt His love, He leaves us all alone; but if we show that sorrow does not make us shrink from Him, He will use the sorrow as the opportunity of showing His tender care for us.

Nothing binds the soul to Jesus so much as outward trouble, for this makes us experience His individual tenderness. We learn therein the lesson of the Cross, and we learn to praise Him for the sufferings which He Himself has borne on our behalf.—*R. M. Benson.*

We must not think we need only to be supported under our affliction. Those who are pressing forwards to a better country will not rest unless they are also sanctified by it; unless each successive wave that passes over them sweeps from their souls some of the dross of earth, and leaves some gift of Heaven in its room, so that the changes and chances of this mortal life shall be ever lifting them further from earth, and bearing ever nearer to the land of everlasting peace.—*Anon.*

Above the tempest wild I hear Him say—
"Beyond this darkness lies the perfect day;
In every path of thine I lead the way."

*Anon.*

## Figures and Tables

**Figure f1:**
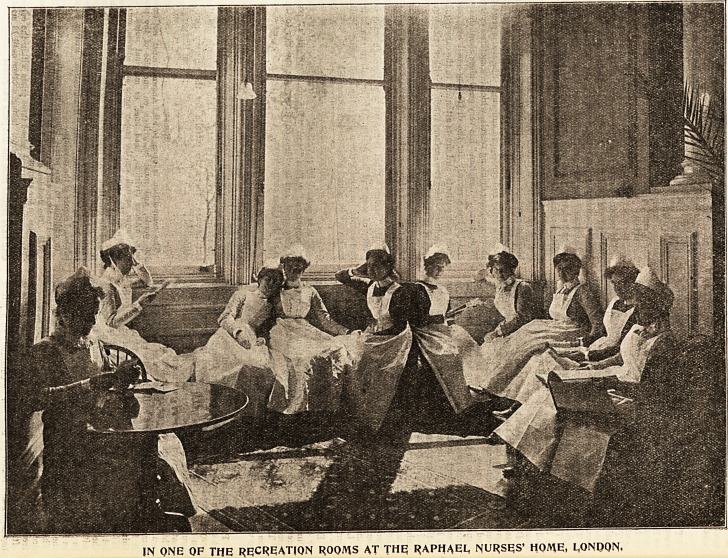


**Figure f2:**